# Hope for brain health: impacting the life course and society

**DOI:** 10.3389/fpsyg.2023.1214014

**Published:** 2023-06-30

**Authors:** Jayashree Dasgupta, Joyla A. Furlano, Zach Bandler, Sol Fittipaldi, Alison J. Canty, Anusha Yasoda-Mohan, Shaimaa I. El-Jaafary, Valentine Ucheagwu, Grainne McGettrick, Vanessa de la Cruz-Góngora, Kim-Huong Nguyen, Brian Lawlor, Aline Nogueira Haas

**Affiliations:** ^1^Global Brain Health Institute, Trinity College Dublin, Dublin, Ireland; ^2^Samvedna Care, Gurugram, India; ^3^Latin American Brain Health Institute (BrainLat), Universidad Adolfo Ibáñez, Santiago, Chile; ^4^Wicking Dementia Research and Education Center, College of Health and Medicine, University of Tasmania, Hobart, TAS, Australia; ^5^Neurology Department, Cairo University, Cairo, Egypt; ^6^Department of Psychology, Nnamdi Azikiwe University, Awka, Nigeria; ^7^Center for Evaluation and Survey Research, National Institute of Public Health, Cuernavaca, Morelos, Mexico; ^8^Center for Health Services Research, Faculty of Medicine, The University of Queensland, Brisbane, QLD, Australia; ^9^School of Physical Education, Physiotherapy and Dance, Federal University of Rio Grande do Sul, Porto Alegre, Brazil

**Keywords:** hope, values, brain health, aging, social brain, scientific education

## Abstract

Hope is a cognitive process by which an individual can identify their personal goals and develop actionable steps to achieve results. It has the potential to positively impact people’s lives by building resilience, and can be meaningfully experienced at both the individual and group level. Despite this significance, there are sizable gaps in our understanding of the neurobiology of hope. In this perspective paper, the authors discuss why further research is needed on hope and its potency to be harnessed in society as a “tool” to promote brain health across healthy and patient populations. Avenues for future research in hope and the brain are proposed. The authors conclude by identifying strategies for the possible applications of hope in brain health promotion within the areas of technology, arts, media, and education.

*“Even if the hopes you started out with are dashed, hope has to be maintained.”*

*–Seamus Heaney, Poet, Nobel Laureate*

## 1. Introduction

The story of humanity has always been driven by an innate capacity for resilience. While many factors underpin the individual’s ability to face and overcome major obstacles, one has remained universally acknowledged regardless of nation or culture: it is the value of hope.

To hope is to be human, and it has been examined in diverse disciplinary perspectives including theology, philosophy, literature, anthropology, and medical sciences. Although there are several definitions of hope in the literature (Schrank et al., 2008), one of the most widely accepted postulates hope as a psychological construct made up of a set of cognitive mechanisms. Specifically, Snyder (2002), defined hope as “*the perceived capability to derive pathways to desired goals, and motivate oneself via agency thinking to use those pathways*.” Agency thinking embodies motivation to initiate and sustain one’s actions to achieve a goal, while “pathway thinking” describes the capacity to find ways to achieve the goal. Simply put, hope is derived from a sense of knowing what one wants to achieve and being able to plan how to get there. While hope overlaps with constructs like optimism and positive thinking, there are subtle differences between these constructs. Optimism is the belief that everything will eventually work out, therefore a passive belief about future outcomes (Alarcon et al., 2013). Positive thinking can be described as a deliberate and conscious effort to manage one’s thoughts, emotions, speech, and beliefs so that they focus only on the possibility of good outcomes during challenging or difficult circumstances (McGrath et al., 2006). Hope, by contrast, is an active process that encompasses both an individual’s perceptions about their ability to plan and strategize, as well as their motivation to follow through with these strategies in a sustained manner, thereby inspiring confidence and enabling decisive action.

Hope is not just an individual experience, but can also be experienced collectively, i.e., a shared vision and belief that planned efforts can be channelized to reach a goal which is important for change (Braithwaite, 2004; Lueck, 2007). Considering the profound potential for hope on the individual, and the ripple effect on the collective predicated by our innate social interconnectedness, it comes as a surprise that so little research and neuroscientific understanding exists on the subject. While theological and philosophical discourse on hope has largely focused around its value to both the individual and larger society, little has been done to practically study its mechanisms in the brain. Could future research be carried out to explore how to “enhance” hope for functional uses and application in the lives of individuals, as well as greater society?

In this perspective, we highlight why understanding hope as a neurobiological entity is an exciting prospect and possible key to tackling larger systemic issues of brain health, and propose directions for future research on hope and the brain.

## 2. Brain networks that may contribute to hope

Since hope is considered a cognitive process, it is reasonable to presume that it may be associated with certain brain networks to our knowledge, two studies have explored this to date.

The first used resting state MRI (rsMRI) to investigate the functional brain architecture underlying hope (Wang et al., 2017). Resting state MRI measures spontaneous brain activity at rest. In this study, the correlational relationship between the intensity of spontaneous brain activity and hope (measured using the Dispositional Hope Scale; DHS) was examined. The DHS is a commonly used 12-item questionnaire that assesses both agency thinking and pathway thinking within the concept of hope. The authors found that higher levels of hope (agency thinking and pathway thinking combined) was related to lower spontaneous brain activity in the bilateral medial orbitofrontal cortex (mOFC). This brain region, located in the frontal lobe, is typically involved in motivation and decision-making processes, which are key elements of hope (Snyder, 1999). Agency thinking and pathway thinking were also independently associated with lower spontaneous brain activity in the mOFC in this study.

In a separate study, Wang et al. (2020) assessed the neuroanatomical basis of hope using structural MRI (sMRI). They examined the association between regional gray matter volumes (GMV) and hope as measured via the DHS. Results showed a positive correlation between hope and GMV of the left supplementary motor area (SMA). The SMA is located in the dorsomedial frontal cortex and is responsible for programming complex movements through linking cognition with action (Cona and Semenza, 2017). Further, it forms a conduit between the prefrontal cortex where higher order thinking and planning occurs, and the primary motor cortex, which initiates voluntary movement (Cona and Semenza, 2017). Since the SMA is involved in goal-directed behaviors, it is no surprise that this area is implicated in hope. Interestingly though, Wang et al. (2020) did not find associations between GMV and the individual components of hope (i.e., agency thinking vs. pathway thinking).

Collectively, these two studies suggest that hope may be associated with networks in the frontal cortex. Further, this area, particularly the prefrontal cortex, has been implicated in concepts closely related to hope including optimism and positive thinking. For instance, Dolcos et al. (2016) showed that optimism is associated with increased GMV in the prefrontal cortex.

## 3. The possible neuromodulation of hope

At the neuromodulator and neurotransmitter level, little is known about hope. However, studies looking at optimism and positive thinking have largely implicated dopamine—a neurotransmitter known to underlie human emotion in the midbrain. Dopamine is important in reward processing and motivation, and may be involved in the anticipation of positive outcomes. One study found that enhancing people’s dopamine function increased their prediction bias in an optimistic direction (Sharot et al., 2012). In this study, participants were administered with a drug that enhanced dopaminergic function or a placebo (control group), and were tasked with providing estimates of their likelihood of experiencing various adverse life events. Those in the experimental group were more likely to make optimistic predictions than the control participants. Given the overlapping components of hope with optimism and positive thinking, there is a high probability that dopamine contributes to the expression of hope. The fact that the mesolimbic system of the brain, which regulates motivation for example, is made up of dopamine-producing cells lends credence to this argument (Yin, 2019).

Other neurotransmitters such as oxytocin, serotonin, and norepinephrine have been implicated in experiencing positive emotions and mood regulation, and thus may also underlie hope. Oxytocin, often called the “love hormone,” is known to be involved in social bonding, and may be associated with feelings of hope and optimism in the context of social relationships (Alexander et al., 2021). Serotonin is believed to be involved in positive emotions including happiness (Matsunaga et al., 2017), and taking serotonin reuptake inhibitors is an effective medication for depression (Michely et al., 2020). It is thought that it may help individuals maintain a hopeful outlook in the face of adversity (Matsunaga et al., 2017). Additionally, norepinephrine plays a role in feelings of motivation and arousal (Sara and Bouret, 2012). In situations where an individual is faced with a challenge or obstacle, the release of norepinephrine may help to increase their sense of determination and hopefulness.

## 4. Future research on hope in the brain

Given that there are only two neuroimaging studies assessing the structural and functional underpinnings of hope, and no studies examining hope on a neuromodulatory or even cellular level, more research in these areas is desperately needed. For instance, functional magnetic resonance imaging (fMRI) could examine functional connectivity patterns of brain regions that may underlie hope. Such research could also explore additional brain regions involved in motivation (such as the amygdala and anterior cingulate cortex; Kim, 2013) and planning (such as the superior colliculus and parietal cortex; Mattar and Lengyel, 2022), as these may also be involved in hope.

In addition, since both neuroimaging studies were correlational in design, we are not able to draw causal conclusions about the neurocircuitry of hope. Thus, studies that are longitudinal and interventional in design are needed. Further, these imaging studies evaluated hope using only a questionnaire. An important step for future studies would be to develop research paradigms, such as task-based approaches for hope, and explore more direct measures or biomarkers of hope. This would allow a more objective investigation of the construct of hope and expand application to other areas of research. Understanding the neurobiological basis of hope also becomes critical at this juncture as we conceptualize symptom-based mental illness models to a more dimensional, transdiagnostic understanding of psychopathology. For example, using frameworks like the research domain criteria (RDoC; Hakak-Zargar et al., 2022) and approaches, which embrace advancements in neuroscience and technology. Task-based hope paradigms could also be used in studies measuring neurotransmitter levels to understand how the two may be correlated.

If future research can unravel the mechanisms and circuitry around hope in the brain, could this be leveraged as an intervention more powerful than pharmacological agents? As discussed above, although current evidence in this area is limited, it is an area of scientific inquiry worth considering. Future research to develop evidence-based interventions that specifically target hope across the lifespan could become a powerful intervention. We discuss this in the context of brain health below.

## 5. Hope to promote brain health

The World Health Organization recognizes brain health^1^ as occurring across cognitive, sensory, social–emotional, behavioral, and motor domains (Brain Health, 2022). Irrespective of the presence or absence of brain-related disease, achieving an optimal level of brain health enables success across the life course (Brain Health, 2022). For example, a recent review highlights how specific components of hopelessness could be considered a clinical target for intervention as it improves psychological flexibility and adaptability (Marchetti et al., 2023).

Further, the individual and collective nature of hope makes it worth investigating further for its potential as a wider reaching “tool.” Hope as a “tool” could help achieve this objective for individuals, communities, and societies, exercised as a type of cognitive training through use in technology, arts, media, and education.

We postulate that if hope can be developed through the life course, or possibly embedded in consumer products and user-based content for the commercial marketplace, perhaps it can be a large strategic contributor to preventative brain health and better living. For example, patients and their families are often devastated following the diagnosis of brain health conditions like dementia, activating a sense of doom that can lead to anxiety, loss, and despair (Aminzadeh et al., 2007; Lawlor, 2021). Higher mortality rates have been shown among caregivers of elderly individuals with disabling conditions (Sullivan, 2003), and hopelessness has been shown to predict mortality in older adults (Zhu et al., 2017). In these cases, instilling hope could be used to improve patient and family experiences.

While psychological interventions like cognitive behavior therapy and supportive counseling are effective interventions for depression where hopelessness is a common clinical feature, unpacking the mechanisms of hope and neurobiological basis offer potential to develop interventions specifically targeting the use of hope in a clinical setting (Cuijpers et al., 2013; Hernandez and James, 2021; Marchetti et al., 2023). Recent years have seen an abundance of mental health apps for “therapy on-the-go,” and while the evidence around their clinical efficacy is limited (Torous et al., 2018; Marshall et al., 2020), they have become incredibly popular among consumers and are increasingly being used as an adjunct. Could future digital applications draw from research on hope in a similar manner to mindfulness and meditation apps, to encourage their users to develop hopefulness? One could envision cognitive training exercises, guided sessions and the accountability of push notifications to encourage regular usage to instill hope and improve a user’s brain health. Could hope be something that is improved with daily practice like mindfulness? And if so, would it provide positive emotional and physiological changes in the user’s mind over the long-term? At this juncture, future research is required to understand what components could be targeted most effectively to enhance realistic hope, which is the balance between false hope (denial that circumstances have changed with an illness/condition) and false hopelessness (inability to be hopeful about an alternative future; Evans, 2019).

Arts, including visual arts, music, and dance, can be used to cultivate hope, given that it is a powerful way to connect people, heal, and find joy. Through arts, people can express themselves, build resilience, and foster positive change (Dunphy et al., 2019; McCrary et al., 2021). For instance, people with Parkinson’s disease experienced benefits in quality of life and well-being after participating in dance classes, and this was associated with improved cognitive function (Hasan et al., 2022). Is it possible that these patients also experienced increased hope and this contributed to the positive changes in brain health? If so, hope through the arts could be used to harness brain health change in patients with neurodegenerative disorders.

Media could also play a larger role in developing hope for brain health. Commercial film and television, print and broadcast journalism, as well as digital or social media are often driven by themes of hope in their narratives. The conflicts within these stories are rife with obstacles that the subjects or protagonists must battle along their journeys, for which hope is a tactical weapon of survival. However, rarely do these industries curate content in a hope-conscious way that encourages viewers to take their lessons learned offscreen and into their own lives. Equipped with greater knowledge of hope’s importance on brain health and its potency, these content creators and providers could become more responsible agents of change in the lives of their many global consumers.

Education provides knowledge and skills to pursue goals and overcome challenges, driving neuroplastic changes in the brain that enhance overall connectivity and brain health. Thus, education could be central to first establishing a sense of hope and subsequently building hope within the individual. To achieve this, new hope-focused pedagogies would need to be established and could be used in a number of environments, whether in formal education, vocational training or workplace wellbeing initiatives. Dissemination of hope as an educative tool across all modalities of information exchange could then result in behavior change at the community level, which might then flow into structural changes at the local and national policy level.

## 6. Final thoughts

In this paper, the authors have described a potential neurobiological basis of hope as a cognitive process, postulated future research directions and, most importantly, explored several strategies for its possible application in brain health. While there is a hypothetical nature to the concept of hope and its uses introduced herein, there is a need to move forward in exploring its actualization regardless of skepticism. An emerging global challenge is upon society in the form of an aging population with increased incidence of dementia and other brain diseases. Public health efforts for earlier detection and research into biomarker-based testing is resulting in more affirmative diagnoses before scalable pharmacological cures are successfully identified. This means there is a need to reframe brain health as soon as possible, and doing so from a perspective of realistic hope can build the resilience necessary for individuals, families, communities and societies to deal with the major challenges ahead.

## Data availability statement

The original contributions presented in the study are included in the article/supplementary material; further inquiries can be directed to the corresponding author.

## Author contributions

JD, JF, and ZB wrote and edited the final version of the manuscript. JD, JF, ZB, SF, AC, AY-M, SE-J, VU, GM, VC-G, K-HN, BL, and AN contributed to conceptualization of the paper and wrote the initial draft of the manuscript. All authors contributed to the article and approved the submitted version.

## Conflict of interest

The authors declare that the research was conducted in the absence of any commercial or financial relationships that could be construed as a potential conflict of interest.

## Publisher’s note

All claims expressed in this article are solely those of the authors and do not necessarily represent those of their affiliated organizations, or those of the publisher, the editors and the reviewers. Any product that may be evaluated in this article, or claim that may be made by its manufacturer, is not guaranteed or endorsed by the publisher.

## Footnotes

^1^Brain Health. WHO (internet). Geneva: 2022. Available from: https://www.who.int/health-topics/brain-health#tab=tab_1.
